# Absence of *Leishmania* spp. DNA in road-killed wild mammals in Southern Brazil

**DOI:** 10.1590/S1984-29612024038

**Published:** 2024-07-22

**Authors:** Julia Somavilla Lignon, Diego Moscarelli Pinto, Mariana Accorsi Teles, Maira Aparecida Christello Trindade, Priscila Rockenbach Portela, Silvia Gonzalez Monteiro, Kauê Rodriguez Martins, Rodrigo Casquero Cunha, Felipe Geraldo Pappen, Bianca Conrad Bohm, Fábio Raphael Pascoti Bruhn

**Affiliations:** 1 Laboratório de Epidemiologia Veterinária, Departamento de Veterinária Preventiva, Universidade Federal de Pelotas – UFPel, Pelotas, RS, Brasil; 2 Laboratório do Grupo de Estudos em Enfermidades Parasitárias, Departamento de Veterinária Preventiva, Universidade Federal de Pelotas – UFPel, Pelotas, RS, Brasil; 3 Laboratório Regional de Diagnóstico, Departamento de Patologia Veterinária, Universidade Federal de Pelotas – UFPel, Pelotas, RS, Brasil; 4 Laboratório de Parasitologia, Departamento de Microbiologia e Parasitologia, Instituto de Biologia, Universidade Federal de Pelotas – UFPel, Pelotas, RS, Brasil; 5 Laboratório de Parasitologia Veterinária, Departamento de Microbiologia e Parasitologia, Universidade Federal de Santa Maria – UFSM, Santa Maria, RS, Brasil; 6 Laboratório de Biologia Molecular Veterinária, Departamento de Veterinária Preventiva, Universidade Federal de Pelotas – UFPel, Pelotas, RS, Brasil

**Keywords:** Leishmaniasis, protozoa, PCR, One Health, zoonosis, Leishmaniose, protozoários, PCR, Saúde Única, zoonose

## Abstract

Leishmaniasis are neglected diseases transmitted by vectors that affect domestic and wild animals, including humans. Due to its incidence and lethality, this zoonosis is a worrying public health problem, making it essential to identify all links in the transmission chain. Infection of wild mammals by *Leishmania* spp. remains poorly understood, especially in southern Brazil. Therefore, the objective was to research, using the PCR technique, the presence of *Leishmania* spp. DNA in road-killed wild mammals in Southern Brazil. Carcasses of 96 animals were collected from highways in the Pelotas microregion, Rio Grande do Sul, southern Brazil and subjected to necropsies. Tissue fragments (spleen, skin, liver, kidney, heart, lung, lymph nodes, bone marrow and blood) were collected and genomic DNA was extracted. PCR protocols targeting the ITS1, kDNA and 18S genes were tested. We found no evidence of *Leishmania* spp. circulation in the studied population. However, epidemiological studies like this one are of great relevance, as they allow monitoring of the occurrence of pathogens and help identify possible risk areas. As these animals act as epidemiological markers for the presence of the microorganism, studies must be carried out continuously to understand whether there are sources of infection in the region.

## Resumo

As leishmanioses são doenças negligenciadas, transmitidas por vetores que acometem animais domésticos e silvestres, incluindo os humanos. Devido a sua incidência e letalidade, essa zoonose consiste em um problema de saúde pública preocupante, sendo fundamental a identificação de todos os elos da cadeia de transmissão. A infecção de mamíferos silvestres por *Leishmania* spp. permanece pouco compreendida, especialmente no sul do Brasil. Portanto, objetivou-se pesquisar, por meio da técnica de PCR, a presença de DNA de *Leishmania* spp. em mamíferos silvestres atropelados no Sul do Brasil. Carcaças de 96 animais foram coletadas, em rodovias da microrregião de Pelotas, Rio Grande do Sul, sul do Brasil e submetidas a necropsias. Fragmentos de tecidos (baço, pele, fígado, rim, coração, pulmão, linfonodos, medula óssea e sangue) foram coletados e o DNA genômico foi extraído. Protocolos de PCR visando os genes ITS1, kDNA e 18S foram testados. Não foram encontradas evidências de circulação de *Leishmania* spp. na população estudada. Porém, estudos epidemiológicos como este são de grande relevância, pois permitem monitorar a ocorrência de patógenos e auxiliam na identificação de possíveis áreas de risco. Como esses animais atuam como marcadores epidemiológicos da presença do microrganismo, estudos devem ser realizados continuamente, para entender se existem fontes de infecção na região.

## Introduction

Leishmaniasis are neglected vector-borne anthropozoonosis caused by infection by parasites of the genus *Leishmania* Ross, 1903 (Kinetoplastida, Trypanosomatidae) (OPAS, 2021; Dias et al., 2022). They are heterogenic parasites whose invertebrate hosts of these protozoa are sandflies, and the vertebrate hosts include various reptiles and mammals (domestic and wild), including humans (OPAS, 2021; Lopes et al., 2023). Furthermore, there is a group of species responsible for maintaining the parasite in nature, called reservoirs. A potential reservoir differs from those that are simple hosts due to the individual persistence of the infection or infectious capacity, that is, the potential to transmit the parasite to vectors (OPAS, 2021).

Considered a complex of diseases, leishmaniasis remains one of the parasitic diseases with the greatest impact on humanity (Lopes et al., 2023). Regardless of the *Leishmania* species, infection by this parasite can be asymptomatic, as well as producing a wide spectrum of clinical manifestations, which can affect the skin, mucous membranes, and viscera. The clinical spectrum of the disease is varied and depends on the interaction of several factors related to the parasite, the vector and the host, however, the visceral form of the disease is the most serious and can lead to death in up to 90% of untreated cases (OPAS, 2021). Given its incidence and high lethality, this zoonosis is a worrying public health problem that mainly affects populations in developing countries, such as Brazil (Lopes et al., 2023), accounting for 93.5% of visceral leishmaniasis cases in the Americas (Melo et al., 2023).

Initially of wild origin and absent in the South of Brazil, leishmaniasis presented important changes in its transmission pattern and is currently found in urban centers and states in the South of the country (Santos et al., 2005; Dias et al., 2022). Some species of *Leishmania* spp. have more restricted circulation in nature. However, many of them have already adapted to urban or peri-urban cycles (e.g., *L. infantum*) (OPAS, 2021) and since 2001 the agent has been present in Rio Grande do Sul (RS), a state considered free of leishmaniasis until then, when the first autochthonous cases of cutaneous leishmaniasis in humans were reported in the cities of Santo Antônio das Missões/RS and Viamão/RS (Santos et al., 2005). In 2006, the first autochthonous case was registered in a canine in the municipality of São Borja/RS (Dias et al., 2022).

Among all new and emerging diseases, around 75% are transmitted from an intermediate animal species to humans (UNEP, 2020) and wildlife can provide a great source of information in little-studied places. Therefore, taking into account the magnitude of the health problem of leishmaniasis and its complex epidemiology, it is essential to identify all links in the transmission chain, in order to implement effective control strategies, which require integrated approaches such as One Health, which focuses on balancing animal, human and environmental health (UNEP, 2020; OPAS, 2021). Understanding each source of transmission allows us to create effective and sustainable strategies for disease surveillance, for this reason, reservoir monitoring has become a focus of concern for public health bodies (OPAS, 2021), and this is necessary for health professionals to have knowledge about the epidemiology of the disease and include it in their clinical suspicions (Dias et al., 2022).

Although several studies demonstrate the infection of wild mammals by *Leishmania* spp., the transmission of these parasites in their natural cycle remains poorly understood, especially in southern Brazil. In this sense, the objective was to research, using the Polymerase Chain Reaction (PCR) technique, the presence of *Leishmania* spp. DNA in road-killed wild mammals in Southern Brazil.

## Material and Methods

### Study area

**Figure 1:**
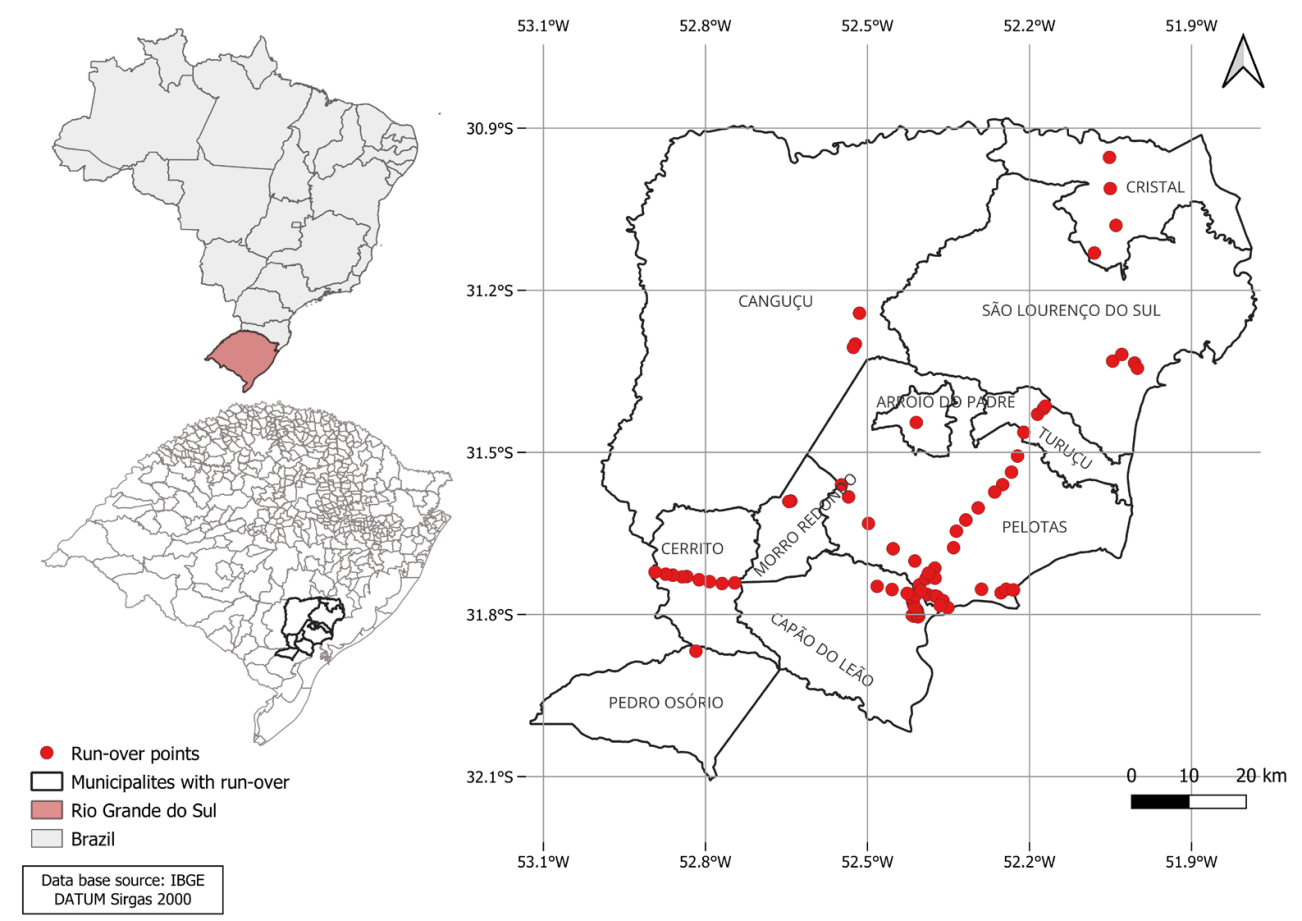
Run-over points where carcasses of 96 road-killed wild mammals were collected in the Pelotas microregion, southern Brazil.

The Pelotas microregion is situated in the south of RS, Brazil, and covers 10 municipalities: Pelotas, Capão do Leão, Pedro Osório, Cerrito, Canguçu, Morro Redondo, Turuçu, São Lourenço do Sul, Cristal and Arroio do Padre (Figure 1). The region covers an area of 10,316,601 km^2^ for a population of 476,096 inhabitants (IBGE, 2023). The climate is subtropical, characterized by well-defined seasons with a high annual temperature range (hot summers and cold winters) in addition to well-distributed rainfall throughout the year. The region comprises the Pampa biome, with fields as the predominant landscape (Roesch et al., 2009). The region has an intense flow of people and animals.

### Animal collection

Carcasses of road-killed wild animals were collected on the highways of cities belonging to the Pelotas microregion. The team traveled to three pre-established routes: Route 1 - Pelotas, Turuçu, São Lourenço do Sul and Cristal; Route 2 – Pelotas, Morro Redondo, Canguçu and Arroio do Padre; Route 3 - Pelotas, Capão do Leão, Pedro Osório and Cerrito. Each route was carried out once a month for one year, from August 2022 to August 2023, with the starting and ending point being the city of Pelotas (31° 46' 34'' S; 52° 21' 34'' O), as described by Caldart et al. (2021). In addition to the driver, two veterinary medical researchers followed the routes, watching both sides of the road. Preferentially, animals with preserved and unexposed viscera were chosen, with an estimated time between one and seven hours. The collected animals were packed in plastic bags, labeled with species, sex, date, city and place where they were found and transported in isothermal boxes with ice to the laboratory of the Grupo de Estudos em Enfermidades Parasitárias at the Universidade Federal de Pelotas, where were necropsied. The species identification of the animals was confirmed according to Reis et al. (2010). Tissue fragments such as spleen, skin, liver, kidney, heart, lung, lymph nodes, bone marrow and blood from all animals were sampled and frozen at –20^o^C until molecular analyses were carried out, as described by Caldart et al. (2021). All study collection points were mapped using the global positioning system (GPS) with the Google Maps mobile application and inserted into the Qgis 2.14.1 software to construct the map (Figure 1).

### DNA extraction

DNA extractions were performed with commercial kits: PetNAD™ Nucleic Acid Co-Prep Kit (GeneReach Biotechnology Corp., Taiwan, China) for blood and ID Gene™ Spin Universal Extraction Kit (ID.Vet, Grabels, France) for tissues, according to the manufacturer's instructions. The DNA samples were quantified in an ultraviolet light spectrophotometer (Thermo Scientific NanoDrop Lite Spectrophotometer, Waltham, Massachusetts, USA), to evaluate their quality by measuring their purity (260 nm/280 nm), using samples ranging from 1.8 to 2.2, and concentration in nanograms per microliter (ng/uL). Furthermore, electrophoresis was performed in 1% agarose gel to confirm the integrity of the extracted material.

### Polymerase chain reaction (PCR)

For the amplification of DNA from parasites of the genus *Leishmania*, three different PCR protocols were tested according to the conditions described in Table 1. Conventional PCR was performed using the primers LITSR/L5.8SF and K13A/K13B, while nested PCR was performed using R221/R332 followed by R222/R333. In the reactions, 2.0 μL of DNA (50 ng/μL) and the mixture containing 2.0 μL of dNTP (2.5mM), 1.0 μL of each primer (10mM), 2.5μL of buffer solution (10X), 1.25 μL of MgCl2 (50 mM), 0.25 μL of Taq DNA polymerase (5U/μL) and 15 μL of ultrapure water were used, totaling 25 μL. As a positive control, DNA from a sample known to be positive for *Leishmania braziliensis* was used, kindly provided by the Veterinary Parasitology Laboratory of the Federal University of Santa Maria. Ultrapure water was used as a negative control. The amplified products were analyzed by electrophoresis in a 1.5% agarose gel, stained with ethidium bromide (0.5μg/mL) and visualized under ultraviolet light. A molecular weight standard of 100 bp was used (Ladder 100 bp 500 µl, Ludwig Biotechnology, Porto Alegre, Rio Grande do Sul, Brazil).

**Table 1:** Main characteristics of the nucleotide sequences used in the PCR in this study.

Primer identification	Primer sequence (5’-3’)	Gene	Product size (bp)	PCR conditions	References
LITSR/L5.8S	CTGGATCATTTTCCGATG TGATACCACTTATCGCACTT	ITS1	314	Denaturation at 94^o^C for 2 minutes, followed by 34 cycles at 95^o^C for 20 seconds, 53^o^C for 30 seconds, 72^o^C for 60 seconds, final extension at 72^o^C for 10 minutes, and 4°C ∝.	Ranasinghe et al. (2015)
K13A/K13B	GTGGGGGAGGGGCGTTCT ATTTTACACCAACCCCCAGTT	kDNA minicircle	120	Denaturation at 94^o^C for 2 minutes, followed by 40 cycles at 94^o^C for 30 seconds, 58^o^C for 30 seconds, 72^o^C for 30 seconds, final extension at 72^o^C for 10 minutes, and 4°C ∝.	Lachaud et al. (2002)
R221/R332	GGTTCCTTTCCTGATTTACG GGCCGGTAAAGGCCGAATAG	18S	603	Denaturation at 94^o^C for 2 minutes, followed by 35 cycles at 94^o^C for 30 seconds, 55^o^C for 30 seconds, 72^o^C for 30 seconds, final extension at 72^o^C for 10 minutes, and 4°C ∝.	Caldart et al. (2021)
R222/R333	TATTGGAGATTATGGAGCTG AAAGCGGGCGCGGTGCTG	18S	358	Denaturation at 94^o^C for 2 minutes, followed by 35 cycles at 94^o^C for 30 seconds, 60^o^C for 30 seconds, 72^o^C for 30 seconds, final extension at 72^o^C for 10 minutes, and 4°C ∝.	Caldart et al. (2021)

## Results and Discussion

In total, carcasses of 96 animals were collected, including two *Hydrochoerus hydrochaeris*, four *Leopardus geoffroyi*, 56 *Didelphis albiventris*, one *Procyon cancrivorus*, five *Cerdocyon thous*, one *Conepatus chinga*, one *Lycalopex gymnocercus*, three *Mazama gouazoubira*, one *Euphractus sexcinctus*, two *Dasypus novemcinctus*, 11 *Cavia aperea*, seven *Galictis cujas*, one *Myocastor coypus* and one *Coendou spinosus*. All blood and tissue samples were negative in the PCR technique.

*Leishmania* DNA has already been detected in wild animals in several regions of Brazil (Richini-Pereira et al., 2014; Paiz et al., 2015; Caldart et al., 2021). However, in the south of the country, more specifically in RS, studies are still considered scarce, although cases in humans and domestic animals have increased over the years in the state (Dias et al., 2022). The Pelotas microregion is considered non-endemic for leishmaniasis, although human cases of visceral and cutaneous leishmaniasis have been reported in recent years in the neighboring city of Rio Grande, RS (Brasil, 2023). Unofficial data indicate the occurrence of human visceral leishmaniasis in the city of São Lourenço do Sul, as well as cases of canine visceral leishmaniasis in Pelotas and São Lourenço do Sul (Dias et al., 2022). Cases of the disease in animals often precede cases in humans, thus epidemiological surveys in non-endemic areas represent an essential tool for conducting epidemiological surveillance of leishmaniasis (OPAS, 2021). Early diagnosis of the disease establishment in municipalities considered non-endemic allows for better planning of preventive actions aimed at avoiding or minimizing problems related to this condition in areas without transmission (OPAS, 2021; Dias et al., 2022). It is important to emphasize that leishmaniasis is not a disease subject to eradication after its establishment and that the costs of prevention are lower than those expended for disease containment, making sentinel surveillance in regions considered non-endemic relevant (Dias et al., 2022), which supports the importance of studying the agent in wildlife.

According to the literature, to date only two studies have been carried out in RS with the aim of detecting the presence of *Leishmania* spp. in land mammals: one involving *C. thous* (Padilha et al., 2021) and another involving *D. albiventris* (Soares, 2019). Both species are considered potential reservoirs of the protozoan (Azami-Conesa et al., 2021; OPAS, 2021), however, marsupials stand out, as they can be infected without showing clinical signs of the disease and the fact that they keep the agent in their organism contributes to the maintenance of the parasite in the environment (Azami-Conesa et al., 2021). Padilha et al. (2021) carried out a serological survey of antibodies against zoonotic pathogens such as *L. infantum* in free-ranging wild canids captured in the Pampa biome, including *C. thous*, but none of the animals sampled showed the presence of antibodies. Despite the use of different techniques, the results corroborate the present work. In contrast, Soares (2019) found a prevalence of 34% (17/50) of this protozoan in *D. albiventris* from cities such as Pelotas and Capão do Leão, in the same region as the present study, using the same diagnostic technique (PCR) and the same region target (18S gene). Likewise, the protozoan has already been detected in opossums in other Brazilian states with varying prevalence (Humberg et al., 2012; Lima et al., 2013).

However, according to Finnie (1986), animals belonging to the genus *Didelphis* are extremely adaptable to most different environments, such as forests and human civilization. Furthermore, they are nomadic animals, making it difficult to define their territory, as they travel long distances and remain in one area for relatively short periods, thus facilitating the spread of pathogens. Therefore, the authors believe that the difference in the results of the present study and the study by Soares (2019) is related to the fact that positive animals may have come from other regions that were already infected, as there are no studies that demonstrate the presence of vectors in the location studied.

Taking into account the rest of the wild animal species collected in the study, amastigote forms, antibodies or *Leishmania* DNA were detected in *D. novemcinctus* in the state of Pará, Brazil (Lainson & Shaw, 1989), in *C. chinga* in Bolivia (Telleria et al., 1999), in *C. aperea*, *P. cancrivorus* and *H. hydrochaeris* in São Paulo, Brazil (Richini-Pereira et al., 2014), in *C. cujas* in São Paulo, Brazil (Paiz et al., 2015) and in *E. sexcinctus* in Rio Grande do Norte, Brazil (Barbosa et al., 2020). We believe that the divergence between the results may be related, here too, to the absence of the vector, since most of these vertebrate species usually travel short distances. Therefore, the specimens analyzed in the study are probably native to this location and not from other regions where leishmaniasis is endemic.

*Leishmania* DNA was not detected in *M. gouazoubira* and *C. spinosus* in the state of Paraná, Brazil (Caldart et al., 2021) and in *M. coypus* in São Paulo (Richini-Pereira et al., 2014) and antibodies against the agent was also not found in *L. gymnocercus* in Minas Gerais (Curi et al., 2006) and Rio Grande do Sul (Padilha et al., 2021), in Brazil. These results are similar to those of the present study for these species. Still, no studies were found on the research of the protozoan in question in *L. geoffroyi* and these specimens appear to be the only ones tested to date. Considering that domestic cats are potential reservoirs of *Leishmania* (OPAS, 2021), understanding whether free-living wild felines participate in transmission cycles is important for a better understanding of this zoonosis in the wild environment and more studies should be carried out.

We found no evidence of *Leishmania* circulation in the studied population. However, epidemiological studies like this are of great relevance as they allow monitoring of the occurrence of pathogens and help identify possible areas of risk. As these animals act as epidemiological markers for the presence of the microorganism, these studies must be continually carried out in order to understand whether there are sources of infection for humans and animals in the region.
